# Age Estimation Based on Computed Tomography Analysis of the Scapula

**DOI:** 10.3390/medicina60040581

**Published:** 2024-03-31

**Authors:** Gokçe Karaman, Ismail Ozgur Can, Yasin Ertug Cekdemir, Oguzhan Ekizoglu, Handan Guleryuz

**Affiliations:** 1 Turgutlu Forensic Medicine Department, Council of Forensic Medicine, 45400 Manisa, Turkey; 2 Forensic Medicine Department, Faculty of Medicine, Dokuz Eylül University, 35220 İzmir, Turkey; ozgur.can@deu.edu.tr; 3 Radiology Department, Faculty of Medicine, Dokuz Eylül University, 35220 İzmir, Turkey; dr_yasincekdemir@yahoo.com (Y.E.C.); handan.guleryuz@deu.edu.tr (H.G.); 4 Unit of Forensic Imaging and Anthropology, University Centre of Legal Medicine Lausanne-Geneva, Geneva University Hospital and University of Geneva, 1015 Lausanne, Switzerland; drekizoglu@gmail.com; 5 Department of Forensic Medicine, Tepecik Training and Research Hospital, 35180 Izmir, Turkey

**Keywords:** scapula, forensic age estimation, computed tomography

## Abstract

*Background and Objectives*: Age estimation from skeletal remains and in living individuals is an important issue for human identification, and also plays a critical role in judicial proceedings for migrants. Forensic analysis of ossification centers is the main evaluation method for age estimation, and ossification degree can be determined using computed tomography analysis. The purpose of this study is to investigate the applicability of CT (computed tomography) in the analysis of left scapula ossification centers, for forensic age estimation in Turkish society. *Materials and Methods*: We analyzed six ossification centers of the left scapula and these ossification centers are the coracoid, subcoracoid, coracoid apex, acromial, glenoid, and inferior angle ossification centers. A pediatric radiologist analyzed these six ossification centers of the scapula by using a staging method defined by Schmeling et al. in 2004. Two months after the first assessment, 20 randomly selected cases was reanalyzed by the first observer and by another pediatric radiologist. Correlation between the age and ossification stage was assessed using Spearman’s nonparametric correlation test. Linear regression analysis was performed using a backwards model. Cohen’s kappa coefficient was used for evaluating interobserver and intraobserver variability. *Results*: In this retrospective study, 397 (248 male and 149 female) cases were evaluated. Ages ranged between 7.1 and 30.9. The mean age was 19.83 ± 6.49. We determined a positive significant correlation between the age and the ossification stages of ossification centers analyzed in both sexes. In each ossification center, except inferior angle, all of the stage 1 and 2 cases in both sexes were under 18 years old. Intraobserver and interobserver evaluations showed that reproducibility and consistency of the method was relatively good. *Conclusions*: The present study indicated that CT analysis of scapula ossification centers might be helpful in forensic age assessment of living individuals and dry bones.

## 1. Introduction and Aim

Identification of a corpse can be important, not only for legal death notification but also ethically, legally and criminally [1]. In cases such as murders, war crimes, and natural disasters, identification is essential. One of the basic steps of the identification process is age estimation. By examining a recovered corpse or skeletal remains, the age of the deceased can be estimated at the time of death, and this helps to narrow the list of missing persons for the purpose of identification [2].

For living individuals, when birth records and official identification information cannot be obtained or are unreliable, age estimation may be required for institutions that handle criminal cases, civil law, and retirement-related transactions. Intensive migration to Turkey and other European countries has caused a significant increase in the number of foreigners who can not fully document their age [1,3]. Due to the increasing demand by judicial authorities, more studies are being carried out for estimating bone age, so that a more accurate age estimate can be made [4]. 

Today, the staging system for evaluating the medial clavicle with computed tomography (CT), developed by Schmeling et al. for age estimation, is widely accepted [5]. The use of staging systems is not limited to the clavicle, and studies have been carried out on many bones in the skeletal system for age estimation using imaging methods. Today, publications state that staging systems used on cross-sectional images obtained by magnetic resonance imaging (MRI) or CT benefit the clavicle and other bones [6,7,8,9,10,11,12,13,14]. In these staging systems, the ossification center of the bones and the appearance of the epiphyseal cartilage is staged using imaging methods, and the person’s age is estimated. The scapula is one of the bones recently examined in this context. 

The scapula is a triangular and flat bone behind the rib cage; there is one on the right and one on the left side of the body [15]. Cartilaginous scapula ossifies from eight or more centers [15]; one in the body, two in the coracoid process, two in the acromion, one in the medial margin, one in the inferior corner, and one in the lower part of the glenoid cavity margin [15]. In ossification centers, fusion is usually completed by 23 years of age [16]. These points make the scapula important for age estimation during the growth period [16]. Although the development of the scapula has already been studied anthropologically on dry bones, there are not enough radiological studies on living individuals. Nougarolis et al. evaluated the ossification centers of the scapula by examining the CT images of 263 thoraces [17]. 

In our study, thorax CT scans were assessed. The ossification centers of the left scapula were staged using the staging system defined by Schmeling et al. The data were evaluated according to age groups and sexes, and the usability of the data obtained from scapula CT imaging in age estimation was investigated.

## 2. Material and Method

This study is retrospective, and thorax tomography images of 397 cases were examined. We accepted the ages and sex of the cases as correct. The ages of cases were between 7 and 30, with 248 males and 149 females.

The left scapula was assessed in the thorax CT scans of the cases. Since the left acromion was not included in the imaging area in 16 cases, the acromion-related evaluations were made with the right scapula. In the thorax tomography of 52 cases, the acromial ossification point could not be evaluated because the acromion in the right and left scapula was not included in the imaging area. The sex of the individuals who had thoracic tomography and their age at the time of the tomography were recorded. 

We noted the absence of tumors, acute trauma, infection, arthritis, or dysplasia in the shoulder region in the cases in our study. The socioeconomic level and the ethnic origin of the cases were not considered because no information was available. While assessing the thorax CT images, no information was shared about the individuals’ identity information and medical conditions. No medical intervention was made, and no medication was applied to the people. For these reasons, obtaining informed consent from the patients was unnecessary. Our research was conducted with the approval of the Non-Invasive Clinical Research Ethics Committee. 

CT images were taken with Siemens brand tomography device with Brilliance 190p 64 detectors. In all of the tomography images, the section thickness was 2 mm, and the overlapping interval was 1 mm. Analysis of tomography images in sagittal, coronal and axial planes was performed by a radiologist who is certified by the European Board of Radiology and specialized in pediatric radiology. One month after the first evaluation, 20 randomly selected cases were evaluated by the same radiologist, and an intraobserver evaluation was performed. To perform interobserver evaluation, 20 randomly selected cases were re-evaluated by another radiologist specialized in pediatric radiology. Neither of the radiologists knew the patient’s identification information or the evaluation of the other radiologist. 

**Figure 1:**
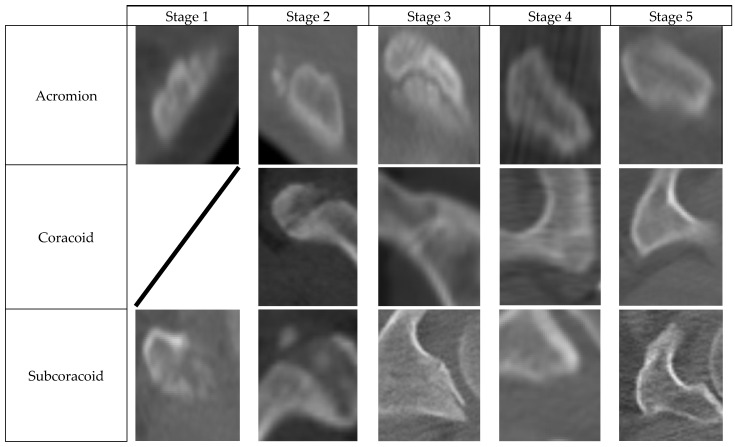

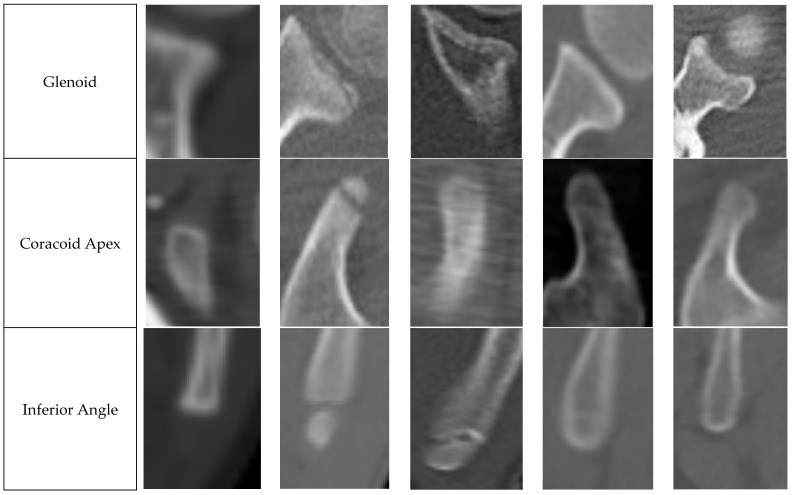
The ossification centers examined in our study.

In our study, 6 ossification centers in the scapula were evaluated. The names of these 6 ossification centers were acromial ossification center, coracoid ossification center, subcoracoid ossification center, glenoid ossification center, coracoid apex ossification center, and inferior angle ossification center (Figure 1).

The 5-stage staging system defined by Schmeling et al. was used for staging these 6 ossification centers. Each of the 6 ossification centers in the scapula was evaluated separately and staged according to the Schmeling method. The staging system used is as follows [5]:

- Stage 1: The ossification center is not ossified.
- Stage 2: The ossification center is ossified, epiphyseal plate is not ossified.
- Stage 3: The epiphyseal plate is partially ossified.
- Stage 4: The epiphyseal plate is completely ossified, and the epiphyseal scar appears.
- Stage 5: The epiphyseal plate is completely ossified, and the epiphyseal scar is not visible.

Statistical analysis was performed using the IBM SPSS 22.0 statistical program. Intraobserver and interobserver evaluations were made using the Cohen kappa coefficient. Descriptive statistical analysis determined the mean age, standard deviation, median value, and 25% and 75% percentile values in each ossification center. Spearman’s nonparametric correlation test was performed at each ossification centers to determine whether there was a correlation between age and ossification stage. To predict age for females and males, linear regression analysis was performed using a backward model.

## 3. Results

Of the 397 cases included in our study, 248 (62.5%) were male, and 149 (37.5%) were female. The youngest case in the study population was 7.1 years old, and the oldest was 30.9 years old. The mean age was 19.832 ± 6.4918 in the entire study population, 20.167 ± 6.4153 in male cases, and 19.276 ± 6.6014 in female cases.

Evaluations in the acromial ossification center (Table 1):

All male and female cases found to be stage 1 were under the age of 15. The youngest female patient, who was found to be stage 2, was 10.9 years old, and the male case was 10.8 years old. The oldest female case found to be stage 2 was 17.8 years old, and the oldest male was 15.5 years old. All of the cases found to be stage 2 were under 18. The oldest female patient, who was found to be stage 3, was 17.7 years old, and the oldest male patient was 20.0 years old. All female cases in which stage 3 was detected were under 18. 

Evaluations in the subcoracoid ossification center (Table 2):

The only female case, which was found to be stage 2, was 10.4 years old, and the male case was 9.1 years old. All male and female cases found to be stage 1 and stage 2 were under 15. The oldest female patient, who was found to be stage 3, was 13.5 years old, and the oldest male patient was 18.2 years old. The oldest female patient with stage 4 was 27.2 years old, and the oldest male patient was 30.3 years old.

Evaluations made at the glenoid ossification center (Table 3):

The youngest female case, which was found to be stage 2, was 10.9 years old, and the youngest male case was 13.2 years old. The oldest female patient, who was found to be stage 2, was 12.3 years old, and the oldest male case was 15.5 years old. All male and female cases found to be stage 2 and stage 3 were younger than 18.

Evaluations made in the coracoid ossification center (Table 4):

The oldest female patient, who was found to be stage 2, was 11.8 years old, and the oldest male case was 12.7 years old. The oldest female patient, who was found to be stage 3, was 13.5 years old, and the oldest male case was 17.2 years old. All male and female cases found to be stage 2 and stage 3 were under 18. The oldest female patient who was found to be stage 4 was 17.6 years old. The oldest male case, who was found to be stage 4, was 24.4 years old.

Evaluations in the coracoid apex ossification center (Table 5):

The oldest female patient, who was found to be stage 2, was 13.7 years old, and the male case was 15.5 years old. Only one female case was identified as stage 3, and she was 13.2 years old. The oldest male case identified as stage 3 was 18.2 years old. The oldest female patient, who was found to be stage 4, was 18.2 years old, and the male case was 24.4 years old.

Evaluations made at the inferior angle ossification center (Table 6): 

The oldest female patient, who was found to be stage 1, was 17.6 years old, and the oldest male patient was 18.2 years old. The youngest female patient, who was found to be stage 2, was 12.5 years old, and the youngest male patient was 13.0 years old. The youngest female patient, who was found to be stage 3, was 13.7 years old, and the youngest male patient was 14.9 years old. The youngest female patient, who was found to be stage 4, was 15.8 years old, and the youngest male patient was 16.3 years old. The youngest female patient, who was found to be stage 5, was 15.4 years old, and the youngest male patient was 16.7 years old. All male and female patients identified as stage 4 and stage 5 were older than 15.

Positive significant correlation was found between age and the ossification stage in the scapula’s ossification centers in both sexes (*p* < 0.001) (Table 7). 

Intraobserver and Interobserver Evaluations:

The thorax CTs of 20 randomly selected cases were re-evaluated by the first observer and another observer who made the evaluations. Intraobserver and interobserver variability were evaluated using the Cohen kappa coefficient. Intraobserver variability was found to be between 0.857 and 1 (Table 8). Interobserver variability was found to bebetween 0.718 and 0.845 (Table 8). 

## 4. Discussion

The coracoid ossification center completes the transition from stage 1 to stage 2 in the first three years of life [18]. The youngest case in our study was 7.1 years old. For this reason, we did not detect stage 1 in the coracoid ossification center. 

It is not appropriate to compare the results of the inspections made on dry bones by Scheuer and Black with the results of our study. On the other hand, since the cross-sectional radiological study data of the scapula is very scarce, it may be important to compare the anthropological data with the data of this study. In the evaluations made on dry bones, it is stated that the ages at which complete fusion occurs in the ossification centers are seen at older ages compared to the evaluations made by radiological methods [19]. In the radiological method used in our study, ossification centers are seen closed at stage 4 and 5. In our study, the ages at which the acromial, coracoid apex and inferior angle ossification centers were found to be closed are earlier than the ages stated by Scheuer and Black. The coracoid and glenoid ossification centers were closed at similar ages. However, our study observed that the subcoracoid ossification center was closed 2 years earlier than the ages stated by Scheuer and Black. Minimal and maximal ages are not specified in the age limits presented by Scheuer and Black. Therefore, the ages at which ossification centers of the scapula are seen and complete fusion in the ossification centers were compared with the median ages determined in our study, and the results are presented below:

- Glenoid ossification center was seen 1 year earlier in our study. The ages at which complete fusion was seen in the glenoid ossification center are similar.
- Subcoracoid ossification center was seen 2 years later in our study. Complete fusion in the subcoracoid ossification center was observed 2 years later in our study.
- The acromial ossification center was seen 2 years earlier in our study. Complete fusion in the acromial ossification center was observed 3 years earlier in our study.
- The ages at which complete fusion is seen in the coracoid ossification center were similar.

The ages at which the coracoid apex ossification center is seen were similar. Complete fusion in the coracoid apex ossification center was observed 4–5 years earlier in our study.

- The ages at which the inferior angle ossification center is seen were similar. Complete fusion in the inferior angle ossification center was seen 3–4 years earlier.

Apart from dry bone analyses, the first radiological cross-sectional study evaluating scapula ossification centers belongs to Nougarolis [17]. Nougarolis analyzed 232 people, 123 males and 109 females, using a 5-stage method over retrospective CT images in French society [17]. Our study basically has a similar study methodology to Nougarolis’ study. For this reason, it will be essential to compare the results of our study with the results of the study of Nougarolis et al. (Table 9). 

In the study conducted by Nougarolis et al., it was seen that male cases detected as stage 4 in the coracoid ossification center were younger than 16 [17]. In our study, male cases over 18 were found to be stage 4 in the coracoid ossification center. 

In the subcoracoid ossification center examined in our study, Stage 3 female cases were observed to be younger than 15, similar with Nougarolis et al [17]. Of the 19 male cases evaluated as stage 3, only 1 was detected at 18.2, and the other 18 cases were younger than 18. 

In the acromial ossification center examined in our study, it was observed that stage 3 female and stage 2 male cases were younger than 18. Nougarolis et al. found that stage 4 male cases were younger than 18, but only 2 were evaluated [17]. 

In the glenoid ossification center examined in our study, stage 1 and stage 2 female cases were younger than 15. Stage 1 and stage 2 male cases are younger than 16. Nougarolis et al. stated in their study that stage 3 female and male cases were younger than 18 in the glenoid ossification center [17]. 

In the coracoid apex ossification center examined in our study, Stage 2 male cases were found to be younger than 18. Only one female case was evaluated as stage 3; she was 13.2 years old. Unlike our study, Nougarolis et al. stated that all stage 3 female and male cases were younger than 18 [17]. 

In the inferior angle ossification center examined in our study, it was observed that all stage 1 female cases were younger than 18. It is seen that stage 1 male cases may be older than 18 years. In the study conducted by Nougarolis et al., unlike our study, it was observed in the inferior angle ossification center that stage 1 female cases may be older than 18 [17]. All male cases evaluated as stage 1 were observed to be younger than 18 [17]. 

In our linear regression analysis, the most effective predictors for age estimation of females were inferior angle, subcoracoid, acromial, and coracoid ossification centers. The most effective predictors for age estimation of males were subcoracoid, inferior angle and coracoid ossification centers (Table 10). 

Schmeling et al. state that the socioeconomic development level of the person is more important in bone development than ethnic origin [20,21]. Low socioeconomic development level causes a deceleration in bone development [22]. If the age of an individual with a low socioeconomic development level estimated by using the reference data obtained from the studies conducted on individuals with a good socioeconomic development level, it is likely to be estimated as less than the actual chronological age. However, since this situation will benefit the person, it does not cause an ethical problem. However, in the opposite case, it should be noted that by estimating the individual’s age as higher than the actual chronological age, erroneous conclusions can be reached that may be a disadvantage to the individual. In our study, the socioeconomic levels of the cases were not evaluated because they were not known. However, the socioeconomic development index stated by the United Nations can provide information about the socioeconomic levels of different countries [7]. Considering that the study conducted by Nougarolis et al. was conducted in France, it is likely that the population we evaluated in our study was at a lower socioeconomic level. However, there is a need for studies to determine the socioeconomic status of societies. The differences between the study conducted by Nougarolis et al. and our study might have been influenced by the socioeconomic level differences between the study populations. 

Over time, secular changes in societies also affect bone development. In the United States, Australia, and Portugal, it is stated that bone development accelerates in men and women due to secular changes [23,24,25]. This situation is shown to be environmental factors such as chemical exposure, dietary changes, and an increased tendency in adipose tissue in children [26,27,28]. Secular changes have been reported to affect skeletal development more in women than men by causing menarche to appear at an earlier age in women and causing women to enter puberty earlier [29]. The scapula is also affected by secular changes. Maranho et al. reported that higher standards of living, including better nutrition and universal healthcare, are associated with an increase in height but also with a slender body in the scapula [30]. The scapula shows a certain degree of sexual dimorphism [31]. It is an important bone for sex diagnosis, and when long bones are unavailable, the scapula is a reliable bone and should be used as an alternative for stature estimation [32,33].

The use of radiology in the age estimation process is controversial. According to a survey recently made by the Forensic Anthropology Society of Europe (FASE), most participants found the use of ionizing radiation for age estimation ethically acceptable and they generally ask for X-rays before proceeding with the age assessment [34]. In a letter to the editor, some authors say that medical radiological methods (which are far less invasive than usually proclaimed) have error ranges but these are better known and quantifiable than those of psychosocial assessments [35]. Therefore, it is unreasonable and counterproductive to proceed to verify age in adolescents without medical and radiological tests. 

Recently, it has been observed that there has been an increasing amount of research on postmortem use and benefits of IT (imaging techniques) [36,37,38,39]. Postmortem CT is routinely applied in some centers to evaluate trauma [39]. Evaluation of the compatibility between postmortem radiological imaging and antemortem images can be used for identification, and postmortem radiological imaging is widely used in anthropological and odontological evaluations [40]. From this point of view, we think that scapular ossification centers can be evaluated with CT in postmortem identification procedures, and the data of our study may be useful for age estimation. In addition, it can be difficult to estimate the age in cases where the whole body cannot be obtained, and a limited number of tissues belonging to the deceased person can be obtained. In such a case, we think that, especially if the scapula can be obtained, it can be evaluated for age estimation by performing CT imaging. 

In studies on forensic age estimation, the image quality of the imaging method used is important. In age estimation studies using CT, slice thickness affects observer evaluations. Mühler et al., in their study, stated that CT evaluations related to age estimation in the medial of the clavicle are affected by the slice thickness [41]. In CT images with 1 and 3 mm slice thickness, the ossification center located medial to the clavicle was evaluated as stage 2, while it was interpreted as stage 3 when re-evaluated on CT images with 5 and 7 mm slice thickness [41]. They stated that even CT images with a section thickness of 1 and 3 mm could cause differences due to the section thickness; therefore, tomography images taken for age estimation should have a cross-sectional interval of 1 mm [41]. Due to the high section thickness, it can be concluded that the ossification center is at a higher developmental stage than it is, and the person’s estimated age may be higher than their actual age [41]. Meijerman et al. also state that the ossification center in a clavicle, which is about to close, can be mistakenly seen as having completed its development due to the loss of details in CT images taken at high section thickness [22]. In our study, although the cross-sectional thickness of CT images was 2 mm since the overlapping interval was 1 mm, good image quality was obtained, and forensic age estimation could be examined. 

For forensic age estimations, observers’ experience in this matter is important and may cause differences in results [42]. Wittschieber et al. emphasize that observer competence has an important effect on staging performed in the ossification center medial to the clavicle [42]. They state that the Cohen kappa (κ) value should not be ignored in studies in order to be an objective indicator of intraobserver and interobserver evaluations, and that the Cohen kappa (κ) value is directly related to the competence of the observer [42]. In our study, the observers who evaluated the thorax tomography of the cases were specialist radiologists with sub-branch training in pediatric radiology. However, the lack of experience in age estimation using the defined Schmeling staging system, specifically in scapula ossification centers, and small sample size at some stages, were limitations of our study. However, the intraobserver variability of our study was tested by the re-evaluation of 20 randomly selected cases by the first observer and it was found to be at a very good level (0.857–1) (Table 8). CT images of 20 randomly selected cases were evaluated by a second pediatric radiologist, and interobserver variability was also found to be at a good level (0.718–0.845) (Table 8). In the study by Nougarolis et al., intraobserver and interobserver variability was also tested, and it was at a better level than our study [17]. The age estimation method used in our study can be used as an objective age estimation method, and is not affected by technical issues. 

## 5. Conclusions

As a result, staging of ossification centers of the scapula on computed tomography images can be conducted with the 5-stage classification system defined by Schmeling et al. As a result of the examination of computed tomography images, a positive significant correlation was found between the stage detected in the ossification centers of the scapula and age. Except for the inferior angle ossification center, it was observed that all stage 1 and stage 2 cases were younger than 18. 

## Figures and Tables

**Table 1:** Minimum and maximum ages, mean age, standard deviation (SD), median age, and 25th and 75th percentiles (Q1, Q3) values were detected in the acromial ossification center.

Stage	Sex	Number of Cases	Mean Age ± SD	Youngest Age–Oldest Age	Q1; Median; Q3
1	Female	19	9.3 ± 1.4	7.1–12.4	8.1; 9.1; 10.7
	Male	37	10.4 ± 1.9	7.6–14.6	8.9; 10.0; 11.8
2	Female	8	13.0 ± 2.1	10.9–17.8	11.6; 12.4; 13.4
	Male	6	13.0 ± 1.5	10.8–15.5	11.9; 13.1; 14.1
3	Female	14	14.4 ± 1.5	12.6–17.7	13.0; 14.3; 15.3
	Male	31	16.3 ± 1.6	13.0–20.0	15.1; 16.3; 17.5
4	Female	9	15.9 ± 1.9	13.7–19.3	14.4; 15.4; 18.0
	Male	12	19.1 ± 4.0	15.1–28.9	16.3; 18.2; 21.4
5	Female	80	23.7 ± 4.3	15.3–30.8	20.5; 24.0; 27.2
	Male	139	24.1 ± 3.8	16.3–30.9	20.9; 24.0; 27.6

**Table 2:** The smallest and largest ages, mean age, standard deviation (SD), median age, and 25th and 75th percentiles (Q1, Q3) values were detected in the subcoracoid ossification center.

Stage	Sex	Number of Cases	Mean Age ± SD	Youngest Age–Oldest Age	Q1; Median; Q3
1	Female	16	9.1 ± 1.3	7.1–11.8	7.9; 9.1; 10.3
	Male	32	9.8 ± 1.5	7.6–12.7	8.5; 9.7; 10.9
2	Female	1	10.4 ± 0	10.4–10.4	10.4; 10.4; 10.4
	Male	7	11.7 ± 1.6	9.1–14.6	11.2; 11.8; 12.3
3	Female	7	11.3 ± 1.6	8.2–13.5	10.7; 11.4; 12.4
	Male	19	13.8 ± 1.7	9.4–18.2	13.1; 13.8; 14.7
4	Female	60	17.2 ± 3.5	10.9–27.2	14.7; 16.8; 19.2
	Male	79	19.2 ± 3.1	13.0–29.0	16.7; 19.7; 20.9
5	Female	65	24.6 ± 4.6	12.5–30.8	22.5; 25.0; 28.4
	Male	111	25.3 ± 3.7	15.0–30.9	22.9; 26.0; 28.5

**Table 3:** Minimum and maximum ages, mean age, standard deviation (SD), median age, and 25th and 75th percentiles (Q1, Q3) values were detected in the glenoid ossification center.

Stage	Sex	Number of Cases	Mean Age ± SD	Youngest Age–Oldest Age	Q1; Median; Q3
1	Female	22	9.8 ± 1.7	7.1–13.5	8.1; 9.2; 11.0
	Male	45	10.6 ± 1.9	7.6–15.5	9.1; 10.5; 12.2
2	Female	4	11.7 ± 0.7	10.9–12.3	11.0; 11.8; 12.3
	Male	5	14.3 ± 0.9	13.2–15.5	13.4; 14.1; 15.2
3	Female	1	13.3 ± 0	13.3–13.3	13.3; 13.3; 13.3
	Male	6	13.0 ± 2.3	8.5–14.7	11.8; 13.6; 14.6
4	Female	34	16.0 ± 2.6	9.3–21.5	14.0; 15.8; 18.2
	Male	53	18.7 ± 3.4	13.0–30.3	16.3; 18.2; 20.3
5	Female	88	23.3 ± 4.9	12.5–30.8	19.5; 24.0; 27.2
	Male	139	24.3 ± 4.0	14.5–30.9	21.4; 24.3; 27.9

**Table 4:** Minimum and maximum ages, mean age, standard deviation (SD), median age 25th and 75th percentile (Q1, Q3) values detected in the coracoid ossification center.

Stage	Sex	Number of Cases	Mean Age ± SD	Youngest Age–Oldest Age	Q1; Median; Q3
1	Female	-	-	-	-
	Male	-	-	-	-
2	Female	16	9.1 ± 1.3	7.1–11.8	7.9; 9.1; 10.3
	Male	34	9.9 ± 1.5	7.6–12.7	8.5; 9.7; 11.1
3	Female	8	11.2 ± 1.5	8.2–13.5	10.4; 11.3; 12.3
	Male	22	13.3 ± 1.7	9.4–17.2	11.8; 13.4; 14.6
4	Female	14	13.8 ± 1.5	12.3–17.6	12.5; 13.5; 15.0
	Male	16	17.5 ± 2.9	13.0–24.4	15.3; 17.2; 20.0
5	Female	111	22.0 ± 5.2	10.9–30.8	17.6; 22.0; 26.7
	Male	176	23.2 ± 4.5	13.0–30.9	19.7; 23.1; 27.5

**Table 5:** The smallest and largest ages, mean age, standard deviation (SD), median age, 25th and 75th percentile (Q1, Q3) values detected in the coracoid apex ossification center.

Stage	Sex	Number of Cases	Mean Age ± SD	Youngest Age–Oldest Age	Q1; Median; Q3
1	Female	23	9.8 ± 1.7	7.1–1.6	8.2; 8.29.3; 11.8
	Male	44	10.5 ± 1.9	7.6–14.6	8.8; 10.1; 12.2
2	Female	5	12.6 ± 1.1	11.3–13.7	11.3; 13.3; 13.6
	Male	4	14.1 ± 1.0	13.1–15.5	13.92; 13.9; 15.1
3	Female	1	13.2 ± 0	13.2–13.2	13.2; 13.2; 13.2
	Male	7	14.8 ± 1.7	13.0–18.2	13.2; 14.7; 15.5
4	Female	10	14.3 ± 2.7	10.7–18.2	11.9; 14.0; 17.3
	Male	10	16.7 ± 3.4	10.8–24.4	15.2; 16.4; 17.7
5	Female	110	22.0 ± 5.1	12.5–30.8	17.6; 22.2; 26.7
	Male	183	23.0 ± 4.6	11.6–30.9	19.7; 23.0; 27.4

**Table 6:** The smallest and largest ages, mean age, standard deviation (SD), median age, 25th and 75th percentiles (Q1, Q3) values detected in the inferior angle ossification center.

Stage	Sex	Number of Cases	Mean Age ± SD	Youngest Age–Oldest Age	Q1; Median; Q3
1	Female	40	11.5 ± 2.7	7.1–17.6	9.1; 11.3; 13.4
	Male	61	11.6 ± 2.5	7.6–18.2	9.4; 11.7; 13.6
2	Female	19	15.4 ± 2.1	12.5–20.0	13.9; 15.2; 16.7
	Male	15	15.7 ± 1.4	13.0–17.6	14.7; 16.1; 16.8
3	Female	7	16.8 ± 1.9	13.7–19.3	15.3; 17.6; 18.2
	Male	14	17.3 ± 1.4	14.9–20.0	16.2; 17.5; 18.4
4	Female	12	19.9 ± 3.2	15.8–27.2	17.5; 19.3; 21.7
	Male	15	20.1 ± 2.5	16.3–25.1	18.3; 19.7; 22.1
5	Female	71	24.7 ± 3.9	15.4–30.8	21.9; 24.7; 28.0
	Male	143	24.5 ± 3.7	16.7–30.9	21.3; 24.3; 28.0

**Table 7:** Correlation between age and ossification stages.

		Acromial	Subcoracoid	Glenoid	Coracoid	Coracoid Apex	Inferior Angle
Age	**Correlation** **Coefficient**	0.822	0.831	0.792	0.737	0.729	0.864
	***p*** **-value**	0.000	0.000	0.000	0.000	0.000	0.000
	**Number (n)**	345	397	397	397	397	397

**Table 8:** Cohen kappa coefficient result of intraobserver and interobserver evaluations.

Ossification Center	Intraobserver (n:20)	Interobserver (n:20)
Acromial	0.925	0.845
Subcoracoid	0.933	0.803
Glenoid	0.857	0.789
Coracoid	0.861	0.718
Coracoid apex	1	0.821
Inferior angle	1	0.790

**Table 9:** Comparison of the results obtained from the study of Nougarolis et al [17]. and our study. (The smallest and largest ages detected for each stage are specified, respectively. F: Female, M: Male).

			Stage 1	Stage 2	Stage 3	Stage 4	Stage 5
Acromial	Nougarolis	F	8.5–10.4	9.8–13.6	9.2–18.9	15.0–16.1	14.6–30.6
		M	7.9–9.4	7.4–14.6	9.2–17.9	16.3–29.6	15.2–30.6
	Our study	F	7.1–12.4	10.9–17.8	12.6–17.7	13.7–19.3	15.3–30.8
		M	7.6–14.6	10.8–15.5	13.0–20.0	15.1–28.9	16.3–30.9
Subcoracoid	Nougarolis	F	8.5–9.9	9.2–10.5	9.2–14.5	11.4–30.5	11.6–30.6
		M	7.4–10.7	8.5–10.8	9.5–15.8	15.7–29.3	15.0–30.6
	Our study	F	7.1–11.8	10.4–10.4	8.2–13.5	10.9–27.2	12.5–30.8
		M	7.6–12.7	9.1–14.6	9.4–18.2	13.0–29.0	15.0–30.9
Glenoid	Nougarolis	F	8.5–12.7	9.2–9.2	11.2–16.6	13.9–19.2	11.4–30.6
		M	7.5–12.9	11.5–12.5	7.9–15.8	15.1–25.2	16.3–30.6
	Our study	F	7.1–13.5	10.9–12.3	13.3–13.3	9.3–21.5	12.5–30.8
		M	7.6–15.5	13.2–15.5	8.5–14.7	13.0–30.3	14.5–30.9
Coracoid	Nougarolis	F	-	8.5–10.7	8.8–14.1	13.6–14.5	11.4–30.6
		M	-	7.4–11.5	10.8–15.2	14.1–15.7	14.6–30.6
	Our study	F	-	7.1–11.8	8.2–13.5	12.3–17.6	10.9–30.8
		M	-	7.6–12.7	9.4–17.2	13.0–24.4	13.0–30.9
Coracoid Apex	Nougarolis	F	8.5–13.6	9.2–12.7	11.2–16.6	13.9–13.9	13.0–30.6
		M	7.4–15.2	9.6–13.6	10.4–15.8	16.6–19.7	15.1–30.6
	Our study	F	7.1–1.6	11.3–13.7	13.2–13.2	10.7–18.2	12.5–30.8
		M	7.6–14.6	13.1–15.5	13.0–18.2	10.8–24.4	11.6–30.9
Inferior angle	Nougarolis	F	8.5–18.4	11.6–17.6	13.2–18.8	18.9–22.3	18.4–30.6
		M	7.4–15.4	13.5–16.9	13.5–20.1	17.7–19.1	18.2–30.6
	Our study	F	7.1–17.6	12.5–20.0	13.7–19.3	15.8–27.2	15.4–30.8
		M	7.6–18.2	13.0–17.6	14.9–20.0	16.3–25.1	16.7–30.9

**Table 10:** Regression analysis of the age (r^2^; Male: 0.814, Female: 0.821).

Sex		Unstandardized Coefficients		Standardized Coefficients	t	*p*	95.0% Confidence Interval for B	
		B	Std. Error	Beta			Lower Bound	Upper Bound
Male	(Constant)	6.657	0.911		7.31	<0.001	4.862	8.452
	Subcoracoid	2.991	0.39	0.625	7.67	<0.001	2.222	3.759
	Coracoid	−1.454	0.484	−0.25	−3.003	0.003	−2.409	−0.5
	Inferior angle	2.097	0.206	0.557	10.198	<0.001	1.691	2.502
Female	(Constant)	6.26	1.381		4.532	<0.001	3.526	8.993
	Acromial	1.446	0.447	0.327	3.237	0.002	0.562	2.33
	Subcoracoid	2.248	0.517	0.421	4.347	<0.001	1.225	3.272
	Coracoid	−1.898	0.743	−0.289	−2.554	0.012	−3.369	−0.427
	Inferior angle	1.886	0.254	0.499	7.431	<0.001	1.384	2.388

Male Age = constant (6.657) + subcoracoid stage × (2.991 + coracoid stage × (−1.454) + inferior angle stage × 2.097. Female Age = constant (6.26) + acromial stage × 1.446 + subcoracoid stage × 2.248 + coracoid stage × (−1.898) + inferior angle stage × 1.886; Stage: Stage of the ossification center (1, 2, 3, 4, 5).

## Data Availability

The data are available upon request from the corresponding author.
